# Use of Cysteamine and Glutaraldehyde Chemicals for Robust Functionalization of Substrates with Protein Biomarkers—An Overview on the Construction of Biosensors with Different Transductions

**DOI:** 10.3390/bios12080581

**Published:** 2022-07-29

**Authors:** Rodica Elena Ionescu

**Affiliations:** Light, Nanomaterials and Nanotechnology (L2n) Laboratory, CNRS EMR 7004, University of Technology of Troyes, 12 Rue Marie Curie, CS 42060, CEDEX, 10004 Troyes, France; elena_rodica.ionescu@utt.fr; Tel.: +33-3-2575-9728; Fax: +33-3-2571-8456

**Keywords:** biomarkers, cysteamine (Cys), glutaraldehyde (GA), biofunctionalization of substrates, biosensors, quartz crystal microbalance (QCM), electrochemistry, optics

## Abstract

Currently, several biosensors are reported to confirm the absence/presence of an abnormal level of specific human biomarkers in research laboratories. Unfortunately, public marketing and/or pharmacy accessibility are not yet possible for many bodily fluid biomarkers. The questions are numerous, starting from the preparation of the substrates, the wet/dry form of recognizing the (bio)ligands, the exposure time, and the choice of the running buffers. In this context, for the first time, the present overview summarizes the pre-functionalization of standard and nanostructured solid/flexible supports with cysteamine (Cys) and glutaraldehyde (GA) chemicals for robust protein immobilization and detection of biomarkers in body fluids (serum, saliva, and urine) using three transductions: piezoelectrical, electrochemical, and optical, respectively. Thus, the reader can easily access and compare step-by-step conjugate protocols published over the past 10 years. In conclusion, Cys/GA chemistry seems widely used for electrochemical sensing applications with different types of recorded signals, either current, potential, or impedance. On the other hand, piezoelectric detection via quartz crystal microbalance (QCM) and optical detection by surface plasmon resonance (LSPR)/surface-enhanced Raman spectroscopy (SERS) are ultrasensitive platforms and very good candidates for the miniaturization of medical devices in the near future.

## 1. Introduction

The level of different biomarkers (nucleic acids, proteins, vesicles, cells) [1,2,3] is essential for human health and widely used either for routine check-up or to monitor the effects of medical treatments and assess tumor regression/progression. In this context, the chemical and robust functionalization of the supports is strongly suitable for a specific and ultrasensitive detection of these biomarkers. An example of a chemical pathway often used for metallic and non-metallic supports is based on cysteamine (Cys)/glutaraldehyde (GA) reagents known as the cross-linking chemistry.

Cysteamine is an alkanethiolate molecule with chemo-sensitizing and radioprotective properties and often used in the treatment of cystinosis and related diseases [4]. However, Cys is intensively used either as a stabilizer for gold nanoparticles (AuNPs) or as a linking agent in the functionalization of solid metallic surfaces for self-assembly monolayers (SAMs) due to its thiol (-SH) and amine (-NH_2_) moieties. For example, when a gold-based or coated substrate is incubated with Cys, its unique thiol group facilities SAM formation at room temperature (RT) through the Au–SH bond. Thus, Cys has been frequently used in the construction of sensitive immunosensors for screening the content of specific biomarkers and drugs in both saline buffers (PBS) and body fluids (serum, urine, saliva, etc.) [5,6]. Specifically, Cys (-NH_2_) moiety provides interaction with the carboxyl (-COOH) groups of antibodies via a covalent bond. Moreover, it was found that Cys dissolved in ethanol instead of water or biological buffers induced homogenous layer formation on supports [7].

**Figure 1:**
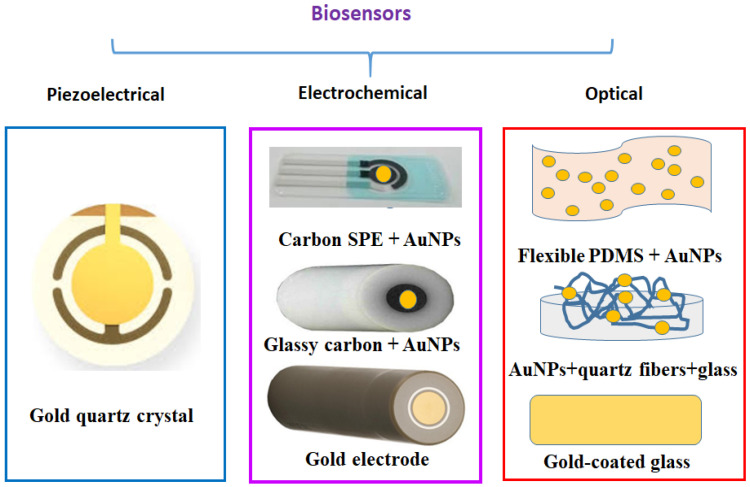
Solid and flexible electrodes used for functionalization with biological molecules and their use for piezoelectrical, electrochemical, and optical biomarker investigations. SPE—screen-printed electrodes; PDMS—poly-dimethylsiloxane.

**Figure 2:**
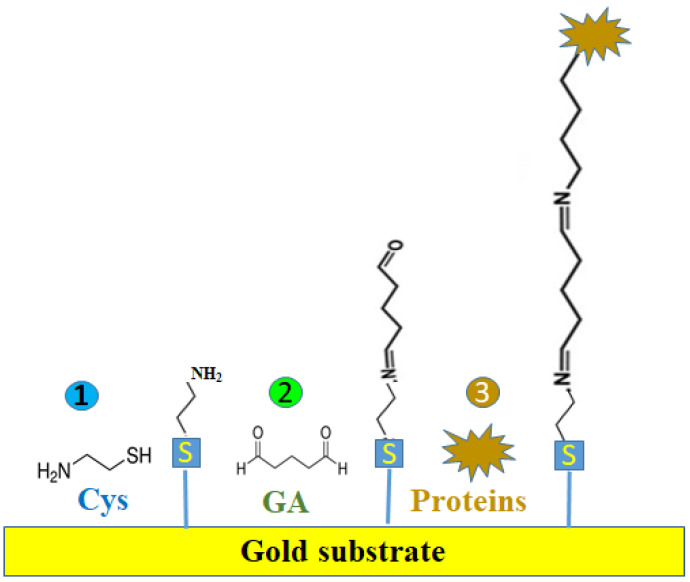
Stepwise immobilization of proteins on the gold substrate using relatively inexpensive cysteamine (Cys)/glutaraldehyde (GA) reagents. Three (bio)functionalization steps are required: (1) incubation with aqueous/ethanol Cys solution; (2) incubation with aqueous GA solution; and (3) incubation with proteins from various human biological fluids such as whole blood, serum, saliva, and urine.

On the other hand, glutaraldehyde is a highly reactive aldehyde reagent, commonly used as a crosslinking agent in several biological tests [8] and very frequently used since 1968 [9]. Thus, its aldehyde group (-CHO) facilitates the covalent immobilization of either larger biological species (e.g., cells, proteins) or small chemical species (e.g., cystea-mine) on various solid/gold nanostructured supports [10] acting as a universal functionalization solution for different transduction methods (Figure 1). For example, if the amino- and sulfur-containing molecules are adsorbed on the gold supports and then activated by GA, its -CHO groups can bind to the –NH_2_ groups of the proteins (e.g., enzymes, antibodies) to form Schiff bases [11,12,13] (Figure 2). Additionally, the excess and physically adsorbed GA is typically removed from supports with ultrapure distilled (DI) water.

As has been widely reported, the use of gold nanoparticles (AuNPs) in the construction of bio-sensors [14,15,16] has been attracting considerable interest due to their large specific surface area, high adsorption capacity, good conductivity, non-toxic nature, and excellent biological quality compatibility. Moreover, a blocking step in the presence of inert biomolecules, such as bovine serum albumin (BSA) containing a high density of lysine residues, are often included in biosensor development before being tested in either a buffered solution or body fluid.

Herein, an overview of the use of the Cys/GA functionalization pathway on various rigid and flexible substrates for the rapid and reproducible detection of various biomarkers with piezoelectrical, electrochemical, and optical transduction is discussed.

Additionally, the step-by-step (bio)functionalization of the electrodes is summarized in tables to facilitate comparison and selection of the most appropriate parameters for future biomarker-based biosensor configurations.

## 2. Piezoelectric Immunosensors

Quartz crystal microbalance (QCM) is a variant of acoustic transducers that under favorable conditions can detect very slight mass changes, in the range 0.1–1 ng/cm^2^. Specifically, a piezoelectric sensor is based on a quartz crystal electrode with two metallic films (typically gold) deposited on both crystal sides. QCM is considered a label-free sensor, where the charge of the mass is measured when adsorbent biomolecules interact with their complementary species immobilized on the electrode surface, therefore increasing the dielectric strength of the crystal and reducing the oscillation of the electrical frequency (Table 1) [17,18,19,20]. Herein, several piezoelectric studies using the Cys/GA reagents are reported and discussed (Table 1).

Homocysteine (Hcy) metabolized in the liver is present in serum samples (>10 μmol/L). The highest values are associated either with a deficiency of vitamins B6 and B12; folic acid; or with the development of cardiovascular diseases, renal failure, and vascular dementia. In this context, the piezoelectric detection on anti-Hcy/silver quartz crystal of two concentration ranges of Hcy (0.1–2 μmol/L and 10–50 μmol/L) with a detection limit of 100 nM Hcy has been reported [21].

Interestingly, by using two-gold QCM sensors modified with either monoclonal anti-human troponin (mAb—cTnT) or used without Ab (as reference electrode) were used to detect the frequency shifts after 14 min of a constant flow of antigen human troponin (cTnT) prepared in PBS (successive injections of 500 pg/mL cTnT-frequency plateau at 4500 pg/mL) or human serum solutions with a LOD of 8 pg/mL [22]. Moreover, the recombinant antigen of *Leishmania chagasi* (rLci2B-NH6) immobilized on quartz crystal (9 MHz) was used for the piezoelectric detection of several dilutions of canine-positive serum (antibodies) up to 1:3200 [23].

*Francisella tularensis*, a Gram-negative bacterium (class 3), induces a zoonotic disease called tularemia that can be transmitted to humans mainly by aerosols. In this context, positive sera (seventh day after infection) were collected from 35 infected European brown hares (Lepus europaeus), and their titer (1:40, 1:80, or 1:160) was confirmed by piezoelectric detection. Interestingly, some discrepancies were observed in the shift frequency and explained by the authors on the basis of the composition of the serum, such as IgG, that induced a five times smaller signal compared to the sample with the same molar concentration of IgM [24].

## 3. Electrochemical Immunosensors

Label-free electrochemical detection of biomolecules either uses the change in the square wave voltammetry (SWV) reduction peak current of a redox probe or monitors the change in real and imaginary impedance (EIS) during immunorecognition events. Moreover, SWV-EIS transductions are considered more sensitive than commercially available ELISA kits [25,26,27,28], while amperometric sensing (current signal measurements) [29,30,31,32] and differential pulse voltammetry (DPV) [33,34,35] are considered advantageous as low-cost technologies and in good agreement with ELISA performance. Herein, several electrochemical studies using the Cys/GA reagents are reported and discussed (Table 2).

A bacterial Gram-positive pathogen *Melissococcus plutonius* that caused the European *foulbrood* (EFB) honeybee diseases was detected with an amperometric GSPE biosensor in the presence of H_2_O_2_/TMB substrate with a LOD of 6.6 × 10^4^ colony-forming unit (CFU) mL^−1^. Moreover, the LODs achieved in the complex matrices of homogenized bees and larvae were 2.4 × 10^5^ and 7.0 × 10^5^ CFU mL^−1^, respectively. It is reported that negative control of *P. alvei* confirmed the high selectivity of such immunoassay [36]. Another study reported on the use of polyimide sheet modified with carbon and AuNPs inks through flexographic printing technique and drop casted with glucoseoxidase (5 μL, Gox, 7 × 10^9^ pg/mL) for chronoamperometry detection of 26 μM glucose mutarotated in DI water for 24 h [37]. On another study, ACE2 (10 × 10^7^ pg/mL) was used in the construction of impedimetric SARS-CoV-2 spike protein immunosensor (0.1 pg/mL to 10,000 pg/mL in PBS buffer and nasal fluid) with 30 min incubation time before recording the EIS spectra [38]. Moreover, gold electrode biofunctionallized with anti-human immunoglobulin and albumin depleted plasma of different dilutions (10^−12^ to 10^−3^) were EIS characterized with a limit of IgG in the range of 4 pg/mL [39]. Gold working electrodes decorated with AuNPs and functionalized with GA/anti-HER-3 antibody were used for EIS and single frequency (500 Hz) impedance detection of HER-3 in artificial serum samples containing 0.2 to 1.4 pg/mL. Interestingly, HER-3 values for healthy people range 60–2550 pg/mL. Unfortunately, in cases of cancer risk (e.g., breast and non-small cell lung carcinoma), the value increases to 12,000 pg/mL [40].

The TSH glycoprotein biomarker responsible for the regulation of human metabolism was detected by EIS (0.1–0.6 mIUL TSH in artificial serum) on a gold electrode modified with polyamidoamine dendrimer (PAMAM) and anti-TSH antibody. The biosensor construction time is 200 min, with good repeatability and no effect of interfering species presented in artificial saliva (AS) [41]. In another study, microcystin with leucine and arginine content (MC-LR), a cyanotoxin that can lead to liver illness/tumor, was impedimetrically detected on glassy carbon electrode (3 mm) modified with AuNPs/Cys/GA/MC-LR-BSA/HRP-mAb + MC-LR. It was found that there was a decrease in EIS spectra in the presence of MC-LR from 10 to 10^5^ pg/mL, with a detection limit of 4 pg/mL (in drinking water accepted MC value by WHO is 10^3^ pg/mL) [42]. Moreover, *Salmonella* Typhimurium (ST), a Gram-negative bacterium causing diarrheic symptoms, fever, abdominal spasm within 12 to 72 h, and occasionally lethal effect, was EIS detected after a heating (80 °C, 40 min)/sonication steps on gold screen printed electrode (GSPE) modified with Cys/GA/monoclonal anti-*Salmonella* antibody. ST was specifically detected in the range 10^3^ CFU mL^−1^ and 10^8^ CFU mL^−1^, while no significant changes of impedance spectra were recorded in the presence of heat-treated interfering *E. coli* K-12 [43].

Otherwise, carcinoembryonic antigen (CEA) was used in the construction of sandwich immunoassays and DPV detected on glassy carbon electrodes successively modified with AuNPs/Cyst/GA/biotinylated anti-CEA antibody/CEA/secondary anti-CEA antibody labelled with HRP molecules. With such sensing configuration, a low limit of quantification (LLOQ) of 7000 pg/mL CEA with a linear range between 1 pg/mL and 5000 pg/mL was obtained. Moreover, in the presence of interference species such as BSA, PSA, CA125 (serum protein found in ovarian cancer), CA 15.3, (serum protein found in breast cancer), and 1 mM Fe (CN)_6_^−3/−4^, no significant current signals changes were observed [44]. Another biomarker, namely, prostate-specific antigen (PSA) in human plasma (0.1 × 10^6^, 10^6^, 2 × 10^6^, 10 × 10^6^, 30 × 10^6^, 60 × 10^6^ pg/mL) was DPV detected on gold electrode modified with electrically generated imprinted polymer based on conducting poly (toluidine blue) (PTB) [45].

DPV technique on synthetic serum and human urine was used to assess the dehydroepiandrosterone 3-sulfate (DHEA−S), a known doping material at different concentrations: 2.5 × 10^3^, 10 × 10^3^, 25 × 10^3^, 50 × 10^3^, 100 × 10^3^, 200 × 10^3^ pg/mL. The authors reported the LOD of 3.97 x 10^3^ pg/mL in working aqueous buffer on a gold electrode modified after anti-DHEA antibody crosslinked with glutaraldehyde. Moreover, two DHEA-S concentrations (25 × 10^3^ and 50 × 10^3^ pg/mL) prepared in synthetic serum and urine were electrochemically tested. Control studies in the presence of nine interferant molecules are reported as well [46].

Three biomarkers of hyper immunoglobulin E syndromes (HIES), namely, activator of transcription 3 (*STAT3*), dedicator of cytokinesis 8 (*DOCK8*), and phosphoglucomutase 3 (*PGM3*) genes were simultaneously detected on carbon electrodes modified with AuNPs by SWV voltammetry with limits of detections of 3.1, 2.2, and 3.5 pg/mL, respectively. These biosensors showed good sensitivity and selectivity against cystic fibrosis transmembrane conductance regulator (CFTR) and Duchenne muscular dystrophy (DMD) [47].

Cyfra 21.1, a soluble fragment of cytokeratin-19, is released during cell apoptosis and highly presented in saliva. Healthy individuals have 3800 pg/mL, while in cancer patients, it is increased to 17,460 ± 1460 pg/mL Cyfra 21.1. With this concern in mind, electrochemical SWV immunosensors using commercial gold working electrodes (Ø = 2 mm) are proposed with 2500 pg/mL Cyfra 21.1 as a low limit of quantification detection in human saliva. Moreover, SWV voltammograms for interfering compounds such as CAE (carcinoembryonic antigen), BSA (bovine serum albumin), PSA (prostate-specific antigen), and their mixture were recorded. It is reported that PSA has no interference in Cyfra21.1 detection, while in the presence of CEA/BSA, either increases or decreases of peak’s currents intensity of Cyfra21.1 were obtained. Moreover, the mixture BSA/CEA/PSA increases the Cyfra21.1 peak’s current that reveals CEA as a dominant interference molecule [48].

Glycodelin (GLY, 47 kDa) protein presented in the bloodstream is a promising biomarker for endometriosis and was successfully detected using a SWV immunosensing platform with a detection limit (LOD) of 430 pg/mL Gly values for healthy women in the range of 5000–31 × 10^3^ pg/mL, while for women with endometriosis is >39 × 10^3^ pg/mL. Moreover, interference compounds, namely, cancer antigens CA 125 (100 U mL^−1^), CA 19–9 (100 × 10^3^ pg/mL), and interleukin 10 with potent anti-inflammatory properties (IL-10, 100 × 10^3^ pg/mL) were mixed with PBS and SWV tested with the GLY (10 ng) immunosensor showed no significant variation in the SWV signals [49]. One year later, leptin, a vital biomarker of non-alcoholic fatty liver (NAFLD), was detected in PBS buffer and human serum by SVW (range 0.150–2500 pg/mL, LOD 0.036 pg/mL) on glassy carbon electrode (3 mm) modified with BP-black phosphorous (BP)/porous graphene (PG)/AuNPs/anti-leptin antibodies (20 × 10^3^ pg/mL). Studies with interference species (100 × 10^3^ pg/mL HSP-70, tumor necrosis factor (TNF)-α, BSA, and interleukin-6 (IL-6)) in the presence of 625 pg/mL showed negligible evolution of SVW signals. When diluted in human serum samples, 100, 500, and 1000 pg/mL leptin were detected by recovery test [50].

NPs of urease were prepared and immobilized on nitrocellulose (NC) membrane pre-coated with chitosan layer wrapped on ammonium ion selective electrode (AISE) and used for the potentiometric detection of urea in human sera from either healthy individuals (3.21 × 10^8^–8 × 10^8^ pg/mL urea) or from patients with kidney pathologies (59.8 × 10^8^–88.4 × 10^8^ pg/mL urea). The detection limit was 1 µmol/L, much lower than the commonly used colorimetric method (0.22 mM) and enzymatic colorimetric method (0.25 mM) with an improved sensitivity 23 mV/decade. Using urea (1 mM) and different interference species, negligible electrochemical signals were recorded in the presence of Na^+^, K^+^, NH^+4^, and Ca^2+^ while Mg^2+^ Cu^2+^, and ascorbic acid modified them slightly [51].

**Figure 3:**
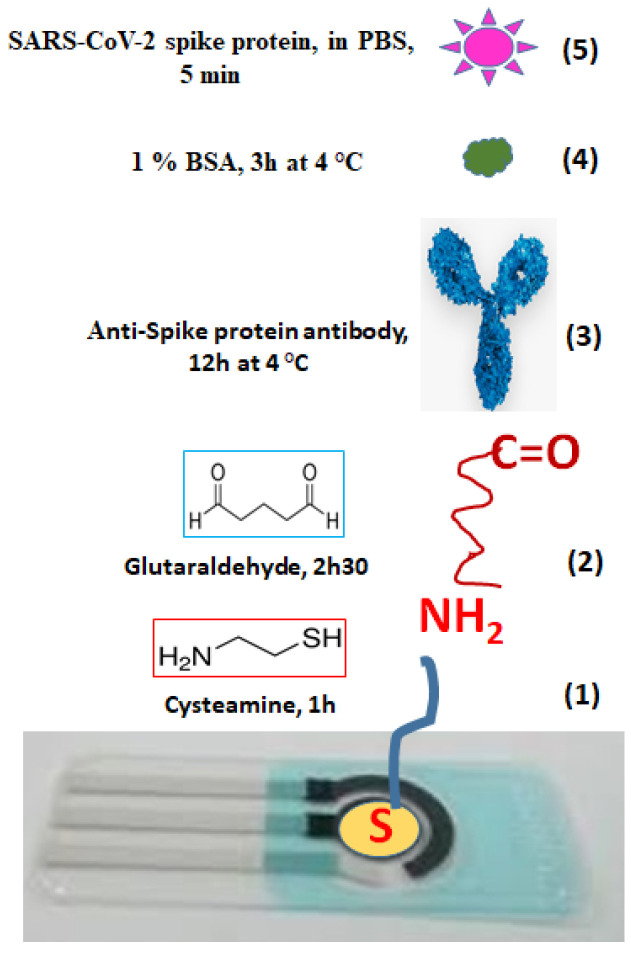
Functionalization of working electrode with AuNPs (yellow circle) and different chemicals cysteamine (1), glutaraldehyde (2), anti-spike protein antibody (3), BSA blocking reagent (4) and SARS-CoV-2 spike protein (5).

Recently, SARS-CoV-2 spike protein (SP) in PBS and in artificial saliva samples was detected on screen-printed electrodes with working electrode (WE, Ø = 3 mm) modified with AuNPs (70–100 nm). It is reported that electrodes cleaned with base piranha (AC) solution provided more intense DPV electrochemical signals and were used for biofunctionalization steps with Cys/GA/anti-spike (10 × 10^6^ pg/mL antibody, 12 µL drop casted) that made the detection of 0.1 × 10^3^ to 500 × 10^3^ pg/mL spike protein possible (Figure 3) [52].

**Table 2:** Step-by-step (bio)functionalization of supports for electrochemical detection of biomarkers on different solid and flexible substrates.

Electrode	Size (Ø)	Cleaning	Cys Activation	GA Activation	Dilution Buffer	Ab	Incu-bation Time/°C (Ab)	Blocking the Non-Specific Sizes	Storage	Ag_2_	Detection Method	Ref.
Au disc (GDE)	1.6 mm	30% H_2_O_2_; conc H_2_SO_4_, 1:3 (*v*/*v*) + polished alumina powder (0.3 and 0.5 μm) + water + ethanol + CV in 0.1 M H_2_SO_4_+ CV in KOH + water + ethanol **(1)**	10 mM Cys in an ethanolic solution for 16 h at 25 °C **(2)**	2.5% GA for 60 min **(3)**	0.05 M PBS, pH 7.4	Anti-GLY Ab 10 × 10^6^ pg/mL 40 min **(4)**	1 h at 37 °C	2% BSA (0.05 M PBS, pH 7.4, for 30 min at 25 °C **(5)**	4 °C in PBS (pH 7.4) (after 30 days) 9% loss for 10 ng GLY Ag **(7)**	GLY protein 10^3^–10^6^ pg/mL for 30 min at 25 °C **(6)**	SWV	[49]
Au	3 mm	SR + Al_2_O_3_ < 50 nm + Drops UPW + UPW + absolute ethanol (99.9%) for 5 min + UPW for 5 min in the ultrasonic + dried with pure Ar **(1)**	100 mM Cys in absolute ethanol for 1 h **(2)**	1% GA for 10 min + 1.5% PAMAM in methanol for 1 h **(3)**	UPW + AS	2.5 ng/anti-TSH for 1 h (5 µL) **(4)**	1 h **(4)**	x	x	0.1–0.6 mIUL^−1^ TSH in artificial serum (AS) **(5)**	EIS	[41]
Au	2 mm	Polished with 0.3 and 0.05 mm alumina slurry + acetone/water (1:1) for 30 min + 0.1 M H_2_SO_4_ **(1)**	10 mM solution Cys in 1 mM ethanol 200 µL, 3 h in dark at RT **(2)**	GA for 30 min RT **(3)**	0.1 M PBS pH 7	Anti-Cyfra 21.1 Ab (50 µL) **(4)**	12 h at 4 °C	BSA for 1 h **(5)**	4 °C	Cyfra 21.1 Ag (2.5, 5, 10, 25, 50) × 10^3^ pg/mL human saliva **(6)**	SWV	[48]
Au	x	0.1 M H_2_SO_4_ + CV + polish with alumina slurry, sized 1.0, 0.3, 0.05 µm **(1)**	100 mM aqueous Cys for 1 h (20 μL) + wash DI water **(2)**	2.5 % GA in WEB (20 μL) + 100 × 10^6^ pg/mL Ab in WEB (20 μL) **(3)**	0.05 M PBS pH 7.4 (WEB)	x	x	x	x	DPV: (2.5, 10, 25, 50, 100, and 200) × 10^3^ pg/mL WEB DHEA−S (10 μL) for 30 min **(4)**	DPV	[46]
Au	x	0.1 M H_2_SO_4_ + 15 CVs **(1)**	10 mM Cys fo1r 1 h + drying **(2)**	GA for 1 h **(3)**	PBS, pH 7.4	x	x	x	4 °C for 24 h	60 CVs for polymerization 0.5 mM TB*^c^ in PBS (pH 7.4) + PSA (1–60) × 10^3^ pg/mL **(4)** + 60 CVs for polymerization 1 M KNO_3_ in PBS (pH 7.4) **(5)** +100 CVs with 0.1 M NaOH **(6)**	DPV	[45]
Au	1.6 mm	0.05 and 0.3 µm alumina + rinsed with ddwater + 0.1 M H_2_SO_4_ + H_2_O_2_/H_2_SO_4_, 1/3 *v*/*v*) for 3 min + ultra-pure water 10× + dry in pure argon + hehexane-dithiol solution (0.1 M in pure ethanol) for 24 h + ethanol + argon **(1)**	10 mM Cys in absolute ethanol for 3 h in dark **(2)**	2.5% GA in water for 30 min (200 µL) **(3)**	PBS, pH 7	10 µg/mL (200 µL) **(4)**	Over-night at 4 °C **(4)**	1% milk 1 h at RT **(5)**	x	Depleted plasma (pg/mL to ×10^6^ pg/mL) for 15 min (20 µL) **(6)**	EIS	[39]
AuNps inks/carbon ink/polyimide sheet + 150 °C for 10 min **(1)**	≈2 mm^2^ electrode with < 60 nm AuNps	Polyimide: ultrasonication with acetone	20 mM Cys for 30 min(5 µL) + ddwater + N_2_ dry **(2)**	4% GA for 30 min (5 μL) + ddwater + N_2_ dry **(3)**	DI water vs. PBS, pH 7.4	x	x	x	x	7 × 10^9^ pg/mL GOx (5 μL) overnight **(4)** …… wash PBS + N_2_ dry **(5)**	ChA	[37]
Au	2.01 mm^2^	Polished with 0.05 μm alumina + ultrasonicaltion in ethanol for 5 min **(1)**	0.5 M Cys in pure ethanol overnight in dark **(2)**	5% GA (5 µL) + 5 × 10^9^ pg/mL anti-HER-3 (5 μL) for 1 h in wet atm **(3)**	Sterile 0.01 M PBS (pH 7)	x	x	1% BSA (10 μL) for 1 h in wet atm **(4)**	Anti-HER-3 and HER-3 solutions at −20 °C	0.2 to 1.0 pg/mL HER-3 solution (5 μL) for 1 h in wet atm **(5)**	EIS	[40]
Carbon + AuNPs by electro-deposition **(1)**	x	x	Cys 2 h at RT **(2)**	2.5% (*v*/*v*) GA in 200 mM PBS (pH 7.4) for 1 h **(3)**	x	Anti-STAT3, anti-PGM3, anti-DOCK8 10 × 10^6^ pg/mL PBS, pH 8.5 **(4)**	1 h **(4)**	0.1 M ethanol-amine for 30 min **(5)**	4 °C wet atm	1 pg/mL to 10^5^ pg/mL STAT3 (for 30 min), PGM3, and DOCK3 for 45 min **(6)**	SVW	[47]
GCE	3 mm	0.3µm and 0.05 µm Al_2_O_3_ slurry + Ultrason (59 kHz, 200 W) with UPW + absolute ethanol + BP (3 μL) + PG (4.2 μL) + IR dried + AuNP solution in dark for 24 h **(1)**	60 mM Cys in pure ethanol + overnight in the dark **(2)**	0.1% GA for 15 min **(3)**	0.1 M PBS, pH 7.4	20 × 10^3^ pg/mL anti-leptin solution (10 μL) **(4)**	In dark for 120 min **(4)**	1% BSA (10 μL) **(5)**	4 °C for 1 week	0.15, 1, 10, 100, 312, 625, 1250 and 2500 pg/mL leptin for 2 h **(6)**	SWV	[50]
Graphite pencils	1 cm lengh (Ø 0.7 mm) **(1)**	Polish sand-paper (2000-grit) **(2)**	AuNP-Cys (pH 7.4) for 75 min **(4)** ………… 50 mM EDC + 25 mM NHS + 10 × 10^6^ pg/mL ACE2 **(5)**	2.5% (*v*/*v*) GA for 1 h at 37 °C **(3)**	0.1 M PBS, pH 7.4	x	30 min at 37 °C	1% BSA (*w*/*v*) for 30 min **(6)**	4 °C dry (stable 24 h) or in PBS (pH 7.4) (stable for 120 h)	SARS-CoV-2 spike protein (SP) **(7)**	SWV	[53]
GCE	Au clusters on GCE after 20 CV cycles of Au solution **(2)**	0.05 µm alumina suspension on felt + water rinsing + ultasonic ethanol/water (1:1) for 5 min **(1)**	20 mM Cys for 1 h (25 μL) **(3)**	7.5% GA in dimethyl formamide for 1 h (25 μL) **(4)**	PBS tablet: 0.01 M PBS + 0.0027 M KCl + 0.137 M NaCl (pH 7.5) at 25 °C	(0.1–1000) × 10^−6^ pg/mL 0.01 M (pH 7.5) PBS solution anti-spike antibody **(7)**	30 min at RT	2% BSA for 20 min **(6)**	4 °C	5 × 10^6^ pg/mL SARS-CoV-2 (2019-nCoV) spike S1-his recombinant protein for 45 min (10 μL) **(5)**	SWV	[54]
GSPE	2 mm	Acetone 15 min **(1)**	20 × 10^9^ pg/mL Cys in water (2 μL) for 2 h at RT **(2)**	5% GA in PBS for 1 h at RT **(3)**	*AB PBS + filtered through 0.22 μm PES mb	0.81 × 10^9^ pg Ab/mL^−1^ PBS **(4)** 0.46 × 10^9^ pg/mLAb-HRP (2 μL) **(7)**	Over-night at 4 °C **(4)** …… 1 h **(7)**	1% BSA in AB for 1 h at RT **(5)**	4 °C (dry electrode with Ab) **(8)**	*Melissococcus* bacteria in PBS (10^5^ to 10^9^ CFU mL^−1^) for 1 h **(6)**	Ampe-rometry + H_2_O_2_/1 mM TMB	[36]
GCE	4 mm	0.3 μm and 0.05 μm alumina slurries + sonication in distilled water and ethanol for 2 min + dry in the air **(1)** ……… HAuCl_4_ solution (1% wt) **(2)**	0.1 M Cys for 12 h at 4 °C **(3)**	2.5% GA for 2 h **(4)**	0.01 M PBS pH 7.4 **(5)**	MC-LR-BSA conjugate 50 × 10^6^ pg/mL (5 μL) **(6)**	6 h at 4 °C **(6)**	0.01 M PBS pH 7.4 + 2 wt % BSA for 1 h at RT (5 µL) **(7)**	Dry at 4 °C **(8)**	10 to 10^5^ pg/mL MC-LR (2.5 μL) + 100 × 10^6^ pg/mL HRP-mAb (2.5 μL) for 40 min at RT **(9)** 1.0 mM 4-CN and 0.15 mM H_2_O_2_, for 15 min at RT **(10)**	EIS	[42]
GSPE	2 mm	Acetone for 20 min **(1)**	20 × 10^9^ pg/mL^−^^1^ Cys in water 2 h **(2)**	3% in PBS for 1 h at RT **(3)**	Filtered PBS, pH 7.4	100 × 10^6^ pg/mL in PBS **(4)**	Over-night at 4 °C **(4)**	BSA in PBS + 0.01% Tween 20 or milk 30 min **(5)**	Dry at 4 °C **(6)**	10^3^–10^8^ CFU/mL Salmonella in tube 1 mL or 10 µL in PBS or milk 15 min RT **(7)**	EIS	[43]
PCB	Formation 75–100 nm AuNPs **(1)**	EC and AC ** (2)	10 mM Cys in absolute ethanol (20 µL) **(3)**	2.5% (*v*/*v*) GA in DI water (10 µL) for 2.5 h **(4)**	1 × filtered PBS *	SARS-CoV-2 spike protein polyclonal Ab (10 × 10^6^ pg/mL, 10 µL) **(5)**	12 h at 4 °C	1% BSA (7 µL) 3 h at 4 °C **(6)**	4 °C **(7)**	Spike protein 0.1 × 10^3^ pg/mL to 500 × 10^3^ pg/mL. 7 µL for 5 min **(8)**	DPV	[52]
NC-mb + 0.2% CHIT (in 2% acetic acid) for 24 h at RT + 10% methanol + 30 min drying **(4)** + urease NPs (0.5 mL) + GA/NC mb overnight at 4 °C “WM” **(6)**	Preparation: urease NPs (ethane/urease = 2:1) 20–100 nm NPs pH 5.5 vs. 13 nm urease pH 7 **(1)**	x	0.12 g Cys under stirring for 5–6 h **(3)**	2.5% GA stirring 500 rpm at 4 °C for 24 h **(2)** …… 2.5% GA in 0.1 M PB, pH 7.3 at RT for 2 h **(5)**	0.1 M sodium acetate buffer, pH 5.5	x	x	x	WM in 0.1 M sodium acetate buffer, pH 5.5, at 4 °C	Urea 2 to 80 µM in 0.1 M sodium acetate buffer, pH 5.5, at 40 °C **(7)**	Poten-tiometry AISE	[51]

Abbreviations: Ab—antibody; Ag—antigen; AISE—ammonia ions selective electrode; BB—blocking buffer; BP—black phosphorous; BSA—bovine serum albumin; CFU—colony-forming unit; ChA—chronoamperometry; CHIT—chitosan; Cys—cysteamine; DHEA—dehydroepiandrosterone 3-sulfate; DPV—differential pulse voltammetry; EIS—electrochemical impedance spectroscopy; EDC—N-(3-dimethylaminopropyl)-N-ethylcarbodiimide hydrochloride; GA—glutaraldehyde, GCE—glassy carbon electrode; GSPE—gold screen printed electrode; NHS—N-hydroxysuccinimide; MC-LR—microcystin with leucin and arginine; RT—room temperature; TMB—3,3′,5,5′-tetramethylbenzidine; BGG—bovine γ-globulin; PCB—printed circuit board modified with 35 μm copper layer/3–5 μm nickel/75–100 nm gold; PG—porous graphene; PVA—poly(vinyl alcohol; SWV—square wave voltammetry; WM—working nitrocellulose membrane; WEB—working buffer. * PBS: 137 mM NaCl, 2.7 mM KCl, 10 mM Na_2_HPO_4_, and 1.8 mM KH_2_PO_4_; PBS—phosphate-buffered saline. ** AC—ammonium hydroxide mixed with hydrogen peroxide for cleaning electrodes in two steps: (i) acetone, ethanol, and DI water (1:1:1) for 20 min and (ii) NH_4_OH:H_2_O_2_:DI water = 1:1:5 for 20 min, providing more DPV electroactive signals when compared with electrodes cleaned with EC—absolute ethanol cleaning for 20 min; GLY—glycodelin; NC—nitroce-llulose. *AB, PBS—assay buffer (pH 7.5): 0.2% BSA, 0.5% BGG, 50 mM Tris, 150 mM NaCl, 5 mM EDTA, 0.2% PVA, 1% glucose, and 0.01% Tween 20; HRP-Ab—anti-*Melissococcus* antibody with HRP, PBS: pH 7.4, 50 mM NaH_2_PO_4_/Na_2_HPO_4_+150 mM NaCl; PAMAM—polyamidoamine dendrimer; SR—synthetic rayon; UPW—ultra-pure water; TSH—thyroid-stimulating hormone. Chronological modification of supports: **(1)** to **(10)** for different biosensing schemes.

**Figure 4:**
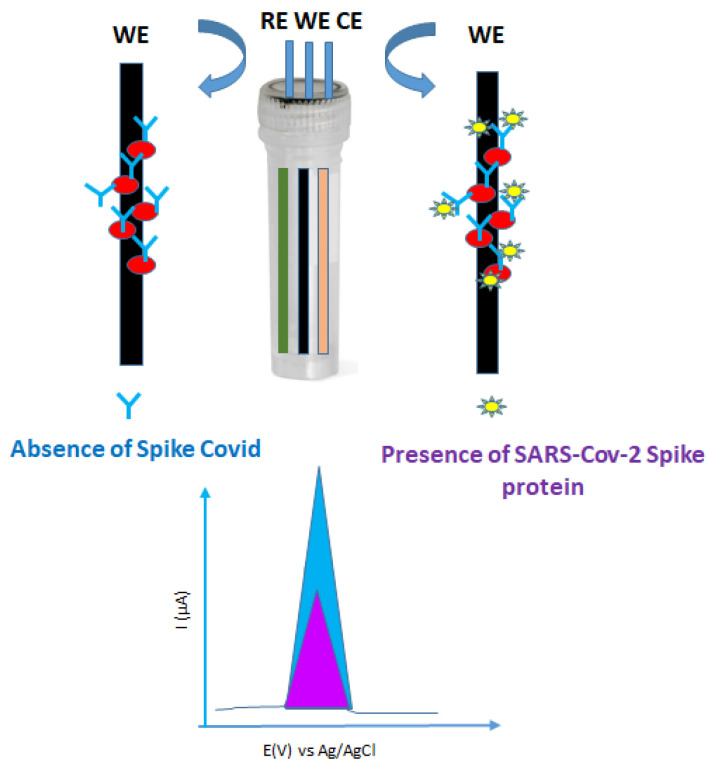
Construction of immunosensor and recorded SWV signals of healthy (absence—blue) and infected (presence—violet) patients with SARS-CoV-2 spike protein.

In another study, graphite pencil electrodes (GPEs) were modified with AuNPs functionalized with Cys-moieties, followed by exposure to an aqueous solution containing EDC/NHS with ACE2 for 30 min at 37 °C. After blocking with BSA at 37 °C for 30 min, the electrodes were exposed for 5 min to different SARS-CoV-2 spike (S1-bis) protein antigen concentrations (range 1-1000 pg/mL), followed by SWVs investigation over 1 min. A LOD of 0.229 pg/mL SP was estimated. Moreover, no cross-reactivity was recorded for four control viral strains: H1N1 (A/California/2009), Influenza-B/Colorado, herpes simplex virus-2, and murine hepatitis virus (MHV) (Figure 4) [53].

Moreover, commercial GCE were modified with AuNPs and used for immobilization of SARS-CoV-2 (2019-nCoV) spike S1-his recombinant protein and SWV tested in the presence of different SARS-CoV-2 spike antibody concentrations (0.1 × 10^−6^–10^−3^ pg/mL PBS (0.01 M, pH 7.5). Moreover, SWV investigations with usual interference species (α-amylase, lipase, Na^+^, K,^+^ Ca^2+^, Mg^2+^, H_2_PO_4_^−^, HPO_4_^2−^, urea) showed no significant changes in the current signal [54].

## 4. Optical Immunosensors

Low-protein biomarker concentrations in the blood in the range of 10^−16^–10^−12^ M are associated with various types of cancer. Therefore, the use of gold nanoparticles (AuNPs) can strongly improve the sensitivity, specificity, resolution, penetration depth, contrast, and speed of the detection of biomarker traces when compared to Western blot and ELISA standard methods [55,56,57]. In this context, Raman optical spectroscopy based on inelastic scattering is defined by the difference in energy between incident photons and vibrational molecules. Moreover, the energy involved in Raman is extremely weak, which is why metallic (e.g., gold and silver) nanostructured substrates are used to amplify the optical signals through the localized surface plasmon resonance (LSPR) phenomenon in the presence of light when the free electrons in the metallic nanostructures are excited, simultaneously inducing collective coherent non-propagating oscillations of surface plasmons [58,59,60]. Moreover, biomarkers have unique spectra, and their identifications in complex biological matrices are expected when using surface-enhanced Raman spectroscopy (SERS). Interestingly, there have only been very few studies on the use of Cys/GA reagents for the functionalization of AuNPs in the construction of optical biosensors, and these are discussed below (Table 3).

One study reports on the adsorption of extracellular vesicles (EVs) and lipoproteins on a quartz microfiber matrix embedded with AuNPs on borosilicate glass after exposure to cysteamine solution. The resulting EVs substrate was used for SERS and SEM investigations using treatment with glutaraldehyde (15 min), osmium tetroxide (15 min), and a series of water/ethanol solutions [61]. Moreover, glass substrates coated with 30 nm AuNPs using the convective self-assembly (CSA) method were used for SERS detection of 4-ABT (LOD 4.7 nM, EF 1.34 × 10^5^) and LSPR detection of IgG (500 nM) with 211% sensitivity improvement vs. continuous gold-coated glass. For both SERS/LSPR investigations, the glass was exposed to 1 mM cysteamine before using the CSA technique [62]. Moreover, unique anti-human IgG antibody concentration (1.5 × 10^6^ pg/mL) was LSPR/SERS detected on Au-coated flexible PDMS film impregnated nanocups incubated with human IgG (10^9^ pg/mL PBS) [63] while human ferritin (0.2 × 10^3^–200 × 10^3^ pg/mL) was detected in human serum using glass biochip based on monoclonal anti-IgM human ferritin (MAbs) immobilized on glass slides and a self-assembled surface plasmon resonance (SPR) system in the Kretschmann configuration [64].

## 5. Conclusions and Perspectives

In this overview, several in vitro biosensing schemes with different transductions (piezoelectrical, electrochemical, and optical) based on Cys/GA chemistry for the immobilization of a specific human biomarker from body fluids are discussed. As an example, among the studies cited, the study on EIS detection of the HER-3 biomarker [40] is particularly useful because it shows the optimization of most effective concentrations such as 0.5 M Cys, 5% GA, and 5 × 10^9^ pg/mL anti-HER3 molecules on supports. Thus, the authors observed that the increasing Cys concentration resulted in a decrease in the charge transfer resistance, while decreasing Cys concentration induced a decay of the electrochemical signal probably due to insufficient formation of SAM by Cys. Even though the resistance values were similar after incubation with 0.1 M, 0.25 M, and 0.5 M Cys, differences in the charge transfer resistance after functionalization with target HER3 molecules were more evident for 0.5 M Cys. Overall, several studies used lower concentrations of Cys such as 1 mM, 10 mM, 18 mM, 20 mM, 50 mM, 60 mM Cys, 100 mM, and 200 mM prepared with different diluents (water or PBS or ethanol/water or absolute ethanol). Additionally, wide variations in incubation time of 30 min, 1 h, 2 h, 3 h, 12 h, 15 h, 16 h/overnight, and 24 h at ambient temperature or 4 °C in the dark or on the bench are noted. Typically, it seems that a 24 h incubation is needed for the lowest 1 mM Cys suspended in water, while the 100 mM Cys is used for either 1 h (diluents: absolute ethanol or PBS) at RT or for 12 h at 4 °C. Contrarily, other study reported the best amperometric detection of p-nitrophenyl phosphate using alkaline-phosphatase-modified, screen-printed gold electrode, which was achieved at 30 °C with glycine buffer (pH 10.5, 50 mM) after 12 h SAM duration [11].

Additionally, GA concentrations in the range of 0.1% to 10% (*v*/*v*) prepared in sterile ultrapure aqueous solution or phosphate-buffered saline (PBS) with incubation times widely dispersed for 15 min to 24 h at either ambient temperature, 37 °C, or 4 °C are reported in Table 1, Table 2 and Table 3. Interestingly, the most commonly reported GA concentration is 2.5% (*v*/*v*), which is in a good agreement with the optimized study on the immobilization of glucose oxidase enzyme on the eggshell membrane for various GA concentrations. The authors claimed that by using 1% to 5% GA, the response of the biosensor increases with the increase in the concentration of GA, while for 5% to 12.5% GA, the biosensor performances (e.g., repeatability, denaturation of enzyme activity) are affected [65]. Moreover, as comprehensively detailed, the process of GA crosslinking with proteins is complex and requires careful optimization for each chosen biomarker target for biosensing investigation (e.g., if the enzyme immobilization is set to 4 °C, long reaction times from 6 h to 18 h are necessary) [66]. Furthermore, under acidic pH conditions, only partial ε-amino groups of proteins are able to react with the aldehyde groups [67], while at alkaline pH values of 7–8, the enzymes are covalently immobilized, and the glutaraldehyde groups have low stability [68].

To date, very few studies have been reported on the clinical simultaneous multiplexed biosensing of multiple biomarkers in large (mL) or tiny (µL) [47] volumes using point-of-care conditions. Fortunately, there are several studies on the preparation of substrates for microarray protein chips with different ending functional groups [69], on the orientation and characterization of immobilized antibodies [70,71], on the proteins/peptides modified hydrogels [72], and on the stability of long SAM layers [73] to greatly support the next generation of point-of-care portable bio devices [74].

Otherwise, to limit non-specific biorecognition events at the surface, exposure to inert proteins such as serum albumin [75] and casein [76] is strongly recommended prior to any biomarker detection at room temperature. Moreover, corroboration with conventional invasive (liquid and tissue biopsy [77]) and non-invasive (e.g., enzyme-linked immunosorbent and immunology-based assays [78] and electrophoretic separation [79]) diagnostic methods with a smartphone-based mobile detection platform are urgently needed for rapid screening between false positive/negative data and to help physicians make an accurate diagnosis of patients before drug treatment and surgical investigations [80,81,82,83,84]. Thus, there is no doubt that low-cost portable cassette sets with pre-treated (e.g., improved Cys/GA chemistry coupled to milder reducing agents and blocking buffers [85,86,87,88]) stable supports based on paper [89,90,91], regular/ultrafine glass [92,93,94], or flexible fiber polymers [95,96] embedded with metallic nanoparticles will continue to attract great interest in the academic and medical [97,98,99,100] research communities.

## Figures and Tables

**Table 1:** Step-by-step (bio)functionalization of supports for piezoelectric detection of biomarkers on different various substrates.

Electrode	Size (Ø)	Cleaning	Cys Activation	GA Activation	Buffer	Ab	Incubation Time/°C (Ab)	Blocking Sites	Storage	Ag	Detection Method	Ref.
Ag-QCM 10 MHz	5 mm	0.5 M NaOH + acetone + methanol for 30 min + DI water + drying at 37 °C for 30 min **(1)**	18 mM Cys in 0.1 M PBS pH 7 for 2 h in dark **(2)**	0.66 M in sodium tetraborate/HCl buffer pH 8.2 for 2 h in dark **(3)**	0.1 M PBS, pH 7.4 (for dil.)	1/10,000 (*v*/*v*) anti-Hcy Ab (3 mL) for 30 min on stirred **(4)**	RT	x	Stock solutions at 4 °C for one week before use	10 μM–50 μM Hcy (3 mL) for 30 min on stirred at RT **(5)**	QCM	[21]
Au-QCM 10 MHz (flow)	8 mm	1:3 mixture of 30%, (*v*/*v*), H_2_O_2_/conc H_2_SO_4_ for 2 min + UPW + ethanol for 5 min **(1)**	25 mM Cys in ethanol for 2 h (static regime) + PBS flow 4 min **(2)**	2.5% (*v*/*v*) GA in 50 mM PBS (pH 7.4) for 45 min (static regime) **(3)**	0.01 M PBS *, pH 7.4	1.2 × 10^6^ pg/mL mAb-cTnT in PBS, (15 μL) in wet condition **(4)**	1 h, 25 °C **(4)**	0.1 M glycine (pH 7.4) for 1 h, in static regime **(5)**	x	cTnT in PBS or serum 800 s (static regime) + PBS wash at flow 100 μL/min for 4 min at 25 °C **(6)**	QCM	[22]
AuQCM 9 MHz	0.8 cm	0.5 M NaOH for 3 min + 3 × washing with ethanol and DI water **(1)**	50 mM Cys in PBS (pH 7.4) for 2 h, at RT **(2)**	2.5% (*v*/*v*) GA for 45 min **(3)**	PBS pH 7.4	Canine serum positive to *L. chagasi* in dilution with 1:3200, 1:1600, 1:800, 1:400 (200 µL) **(6)**	15 min **(6)**	50 mM glycine **(5)**	4 to 8 °C	3 × 10^6^ pg/mL rLci2BNH6 antigen for 1 h **(4)**	QCM	[23]
Au-QCM 10 MHz flow	5 mm	Acetone for 30 min + drying **(1)**	10 × 10^9^ pg/mL Cys for 2 h (20 μL) **(2)**	3% GA in water for 2 h **(3)**	Wash: PBS/0.5% Triton x 100/PBS **(7)** **…………** 0.1 M glycine buffer of pH 2.2 with 0.5% Triton x 100 **(8)**	Sera sample for 10 min (20 μL) **(6)**	RT	10 × 10^9^ pg/mL BSA **(5)**	x	1 × 10^9^ pg/mL Ag (lipid fraction from liver cells) + overnight at 4 °C (20 μL) **(4)**	QCM	[24]

Abbreviations: Ab—antibody; Ag—antigen; cTnT—human cardiac troponin T; Hcy—homocysteine; mAb-cTnT—mouse monoclonal antibody against cTnT; * PBS: 0.2 g KCl, 8.0 g NaCl, 0.24 g KH_2_PO_4_, 1.44 g Na_2_HPO_4_, in 1000 mL UPW; chronological modification of supports: **(1)** to **(10)** for different biosensing schemes.

**Table 3:** Step-by-step (bio)functionalization of supports for optical detection of biomarkers on different solid and flexible substrates.

Electrode	Size Ø	Cleaning	Cys Activation	GA Activation	Dilution/Washing Buffer	Ab	Incubation Time/°C (Ab)	Blocking Sites	Storage	Ag/Analyte	Detection Method	Ref.
AuNPs on quartz fibers	40–60 nm	x	20 mM Cys in 95% ethanol for 1 h (10 μL) **(1)**	GA for 15 min (for SEM)	UPW **(2)**, **(4)**	x	**x**	x	**x**	100 × EV in UVW for 2 h at RT (40 μL) **(3)**	SERS	[61]
NSF10 glass	5 nm Ti + 45 nm Au **(2)**	Sonication in acetone/ethyl (10 min) + rinsed DIW + ethyl alcohol (5 min) + N_2_ drying **(1)**	1 mM Cys for 24 h + 30 nm AuNPs at 50 °C to obtain 5OD **(3)**	x ……. GA 30 min **(4^LSPR^)**	x	500 nM IgG + ethyl alcohol and distilled water for 10 min **(5^LSPR^)**	x	x	x	4-ABT 10^−8^ to 10^−4^ M for 30 min + ethyl alcohol and distilled water for 5 min **(4^SERS^)**	SERS & LSPR	[62]
Au filmed PDMS	1 cm^2^	Glass slide: UV ozone for 20 min + PS + PDMS + 1 h at 60 °C Pelled off PDMS + DMF Coating: 50 nm Au **(1)**	0.2 M Cys aqueous solution in dark at RT for 15 h **(2)**	4% GA at RTfor 4 h **(3)**	PBS pH 7.4 **(4)**	Anti-human IgG 1.5 × 10^6^ pg/mL (50 μL) **(7)**	4 h **(7)**	5 × 10^9^ pg/mL of BSA in PBS for 1 h **(6)**	x	1 × 10^9^ pg/mL human IgG in PBS (pH 7.4) at 20 °C for 15 h **(5)**	LSPR & SERS	[63]
Glass slide + 5 nm Cr + 50 nm Au	**x**	1.2 M NaOH for 10 min + 1.2 M HCl for 5 min + one drop of HCl for 30 s **(1)**	10 mM Cys in 50 mM PBS, pH 7.0, for 1 h + DI + PBS + dry **(2)**	10% GA (*v*/*v* eau) for 30 min + DI wash **(3)**	PBS * + DI water + dry **(6)**, **(8)**	1 × 10^9^ pg/mL anti-ferritin MAbs **(4)**	1 h **(5)**	0.1 M glycine in 50 mM PBS pH 7.0 for 30 min **(7)**	Signal stability for 15 days	Human ferritin 0.2 × 10^3^–200 × 10^3^ pg/mL for 30 min (3 µL) **(9)** …… 0.1 M HCl buffer, pH 2.1 **(10)**	SPR	[64]

Abbreviations: DMF—dimethylformamide; EV—extracellular vesicles; SEM—scanning electron microscopy; OD—optical density; 4-ABT—4-aminobenzenethiol; * PBS composition: 5 mM Na_2_HPO_4_/NaH_2_PO_4_, 0.15 M NaCl; pH 7.0. Chronological modification of supports: **(1)** to **(10)** for different biosensing schemes.
